# Bilateral Inguinal Hernia With Psoas Muscle Involvement: A Case Report and Literature Review

**DOI:** 10.7759/cureus.105240

**Published:** 2026-03-14

**Authors:** Marco Antonio Urbina Velázquez, Olga L Hernández Sosa, Ana Jiménez Martínez, Regina Ramírez Arroyo, Atziri de León Oliva, Emmanuel Calderón Guerrero, Brenda D Chávez Álvarez, Natalia Velázquez Velázquez, Regina C Aguirre Hernández, Regina Tapia García, María Fernanda Aguirre Quintero, José Emiliano González Flores

**Affiliations:** 1 Department of Colon and Rectal Surgery, Centro Médico ABC, Mexico City, MEX; 2 School of Medicine and Health Sciences, Instituto Tecnológico y de Estudios Superiores de Monterrey (ITESM), Mexico City, MEX; 3 Department of Surgery, Instituto Tecnológico y de Estudios Superiores de Monterrey (ITESM), Mexico City, MEX

**Keywords:** atypical groin hernia, bilateral inguinal hernia, inguinal hernia, laparoscopic hernia repair, occult inguinal hernia, posterior compartment hernia, preperitoneal space, psoas muscle, retroperitoneal hernia, tapp repair

## Abstract

Inguinal hernia is one of the most common conditions in general surgery; however, atypical variants extending beyond the conventional myopectineal boundaries are infrequently encountered. Herniation involving the psoas muscle is exceptionally rare due to its deep retroperitoneal location and is often identified only during surgical exploration. We report the case of an 86-year-old woman who presented with persistent right inguinofemoral pain without a palpable groin mass. Preoperative evaluation, including prior cross-sectional imaging, did not demonstrate a definitive inguinal defect. Owing to sustained symptoms and clinical suspicion, elective laparoscopic transabdominal preperitoneal (TAPP) repair was performed. Intraoperative exploration revealed an unusual deep hernia tract on the right side extending posteriorly into the retroperitoneal plane adjacent to the psoas muscle. On the contralateral side, protrusion of preperitoneal fat without a well-defined hernia sac resulted in an asymmetric bilateral presentation. Careful reduction of the retroperitoneal extension and contralateral preperitoneal tissue repositioning were followed by bilateral mesh reinforcement. The procedure was completed without complications. At one-month follow-up, the patient remained asymptomatic with no evidence of recurrence. This case underscores the potential diagnostic limitations of routine preoperative assessment in atypical groin pathology and highlights the value of systematic posterior preperitoneal exploration during minimally invasive repair. Recognition of occult retroperitoneal extension is essential to prevent inadvertent neurovascular injury and ensure adequate mesh coverage. Reporting this rare bilateral presentation with psoas-related involvement expands the recognized anatomical spectrum of inguinal hernia disease and contributes to the limited body of literature on deep posterior variants.

## Introduction

Inguinal hernia is among the most prevalent conditions in general surgical practice and represents a leading indication for elective surgery worldwide [1]. The lifetime risk of undergoing inguinal hernia repair is estimated at up to 27% in men and approximately 3% in women, accounting for millions of procedures annually and reflecting a substantial clinical burden [1]. Inguinal hernia results from a defect in the abdominal wall that allows protrusion of intra-abdominal contents through the inguinal canal. However, uncommon anatomical variants have been described in which the defect extends into deeper planes or involves retroperitoneal structures, potentially hindering both clinical and radiologic identification and remaining undetected until surgical repair [1,2].

Among these atypical anatomical variants, hernias involving the psoas muscle have been reported as exceptionally rare entities that, owing to their deep location and relationship with the retroperitoneal compartment, are most often diagnosed incidentally during the repair of apparently straightforward inguinal hernias [3]. From an anatomical standpoint, the proximity of the psoas muscle to the deep inguinal ring and to the preperitoneal fascial planes provides a structural substrate that may facilitate these unconventional hernia extensions. Interest in the psoas muscle has increased in recent years, not only because of its anatomical relationship with the inguinal region, but also due to its functional relevance as an indirect marker of sarcopenia. Reduced psoas muscle volume has been shown to correlate significantly with an increased risk of inguinal hernia development, suggesting that loss of deep muscular mass may compromise posterior wall integrity and abdominal wall support [4]. In this context, the laparoscopic approach has proven particularly valuable for both the diagnosis and management of complex or non-conventional inguinal hernias, as it enables detailed exploration of the preperitoneal and retroperitoneal spaces, facilitates identification of deep defects, and optimizes prosthetic mesh placement [2,5].

Despite advances in surgical techniques and the growing number of isolated reports, the available evidence regarding inguinal hernias involving the psoas muscle remains limited and is predominantly derived from case descriptions, precluding determination of their true incidence, the establishment of consistent preoperative diagnostic criteria, or characterization of their long-term clinical behavior [2,3]. Furthermore, bilateral presentations and their potential functional implications have been sparsely documented in the literature. Given the limited evidence available, reporting a bilateral inguinal hernia with psoas-related retroperitoneal extension identified intraoperatively is clinically relevant, as it expands the spectrum of atypical posterior groin hernias and informs diagnostic and operative considerations. To the best of our knowledge, bilateral inguinal hernia with extension toward the psoas muscle has been rarely described, underscoring the anatomical and clinical uniqueness of the present case. This report aims to describe a rare case of bilateral inguinal hernia with psoas muscle involvement and to contextualize this finding within the existing literature, highlighting its diagnostic and surgical relevance.

## Case presentation

An 86-year-old woman was referred for evaluation of right inguinofemoral pain. She was independent in daily activities and able to ambulate several blocks without assistance. Her medical history was notable for systemic arterial hypertension adequately controlled with losartan. No additional relevant comorbidities were reported. She had no prior abdominal surgery.

The patient described localized pain in the right inguinal region, characterized by exacerbation during ambulation and when climbing stairs. She also reported a sensation of tightness in the right groin, particularly after prolonged standing or physical exertion. Symptoms were associated with activity-related discomfort of the right lower limb, which improved with rest. Additionally, she noted a progressive perception of discomfort in the inguinal region over recent months, which had begun to interfere with her usual mobility. Functionally, the condition had resulted in a recent limitation of her domestic activities.

On physical examination, the right inguinal region revealed localized tenderness without a palpable mass, skin discoloration, or signs suggestive of incarceration. Pain was elicited on deep palpation of the right inguinal canal, raising clinical suspicion of an underlying inguinal wall defect. Examination of the left inguinal region revealed no clinically relevant abnormalities.

Regarding imaging studies, a prior non-contrast computed tomography scan (performed months earlier for an unrelated indication) had not demonstrated inguinal wall defects or retroperitoneal abnormalities relevant to the current presentation. Based on persistent symptoms and clinical assessment suggestive of an inguinal hernia, the patient was referred for specialized surgical evaluation.

Following positive clinical correlation, elective surgical management was planned in accordance with institutional protocols. A laparoscopic transabdominal preperitoneal (TAPP) hernia repair with mesh placement was performed.

**Figure 1:**
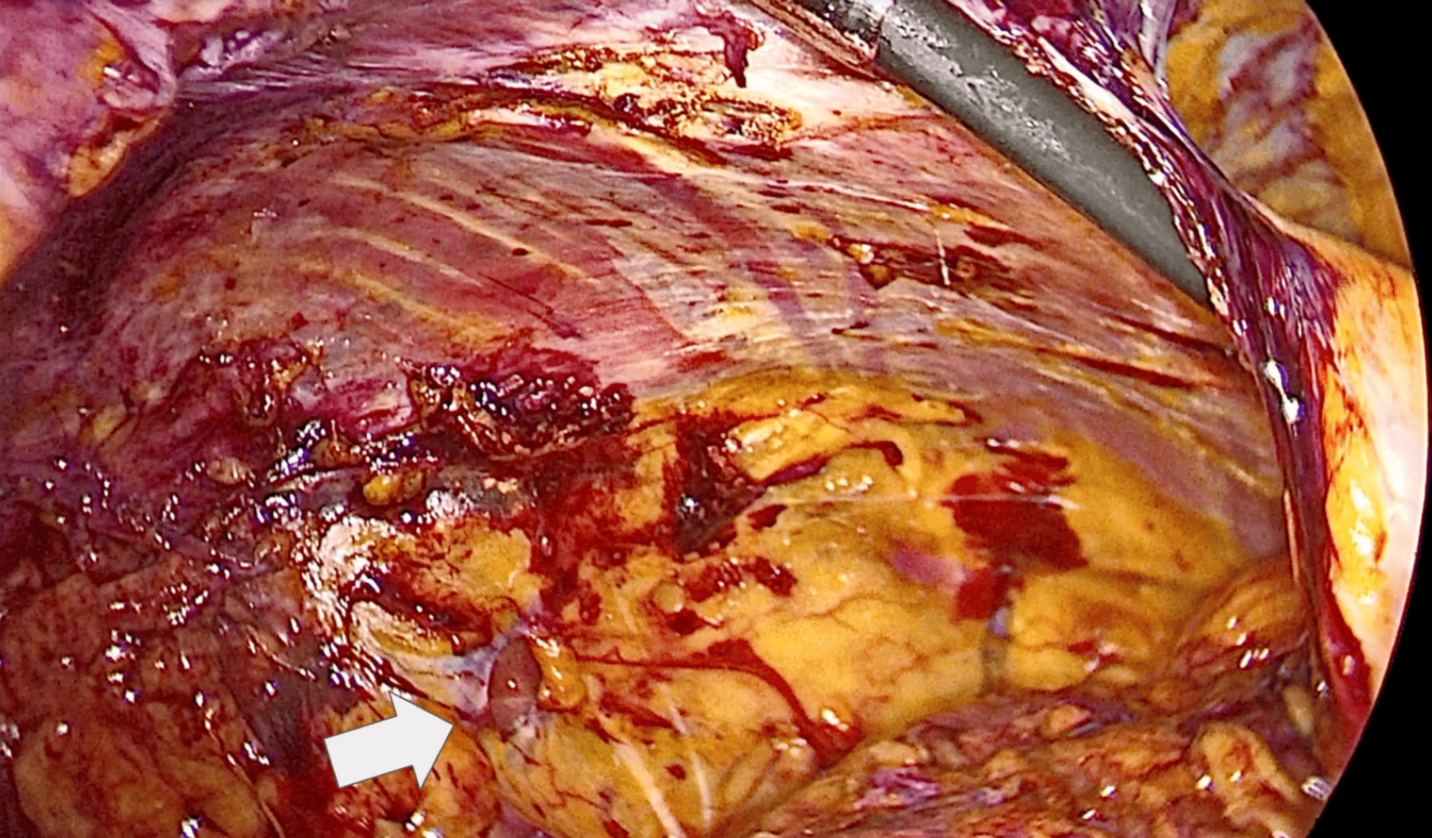
Deep right-sided hernia trajectory extending toward the psoas muscle compartment Intraoperative laparoscopic view demonstrating a deep right-sided hernia trajectory extending toward the psoas muscle compartment (arrow). The protrusion of preperitoneal adipose tissue into the posterior plane highlights the unusual retroperitoneal extension not identified on preoperative imaging.

**Figure 2:**
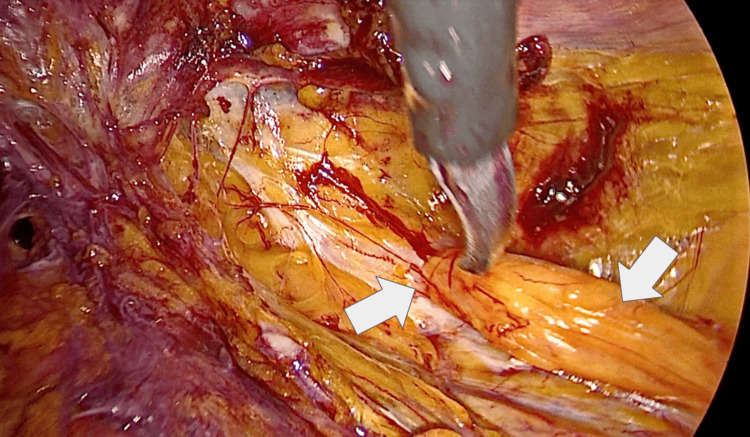
Contralateral protrusion of preperitoneal adipose tissue without defined hernia sac Intraoperative laparoscopic view demonstrating the hernia defect (left arrow) with protrusion of preperitoneal adipose tissue (right arrow) in the absence of a well-defined hernia sac. This finding contrasts with the deep retroperitoneal extension observed on the contralateral side and illustrates the asymmetric bilateral presentation.

Intraoperatively, the right side revealed a deep hernia sac extending toward the retroperitoneal psoas compartment, representing an unusual anatomical localization not identified on preoperative imaging (Figure 1). On the left side, protrusion of preperitoneal fat was observed without a clearly defined hernia sac, illustrating an asymmetric bilateral presentation (Figure 2).

**Figure 3:**
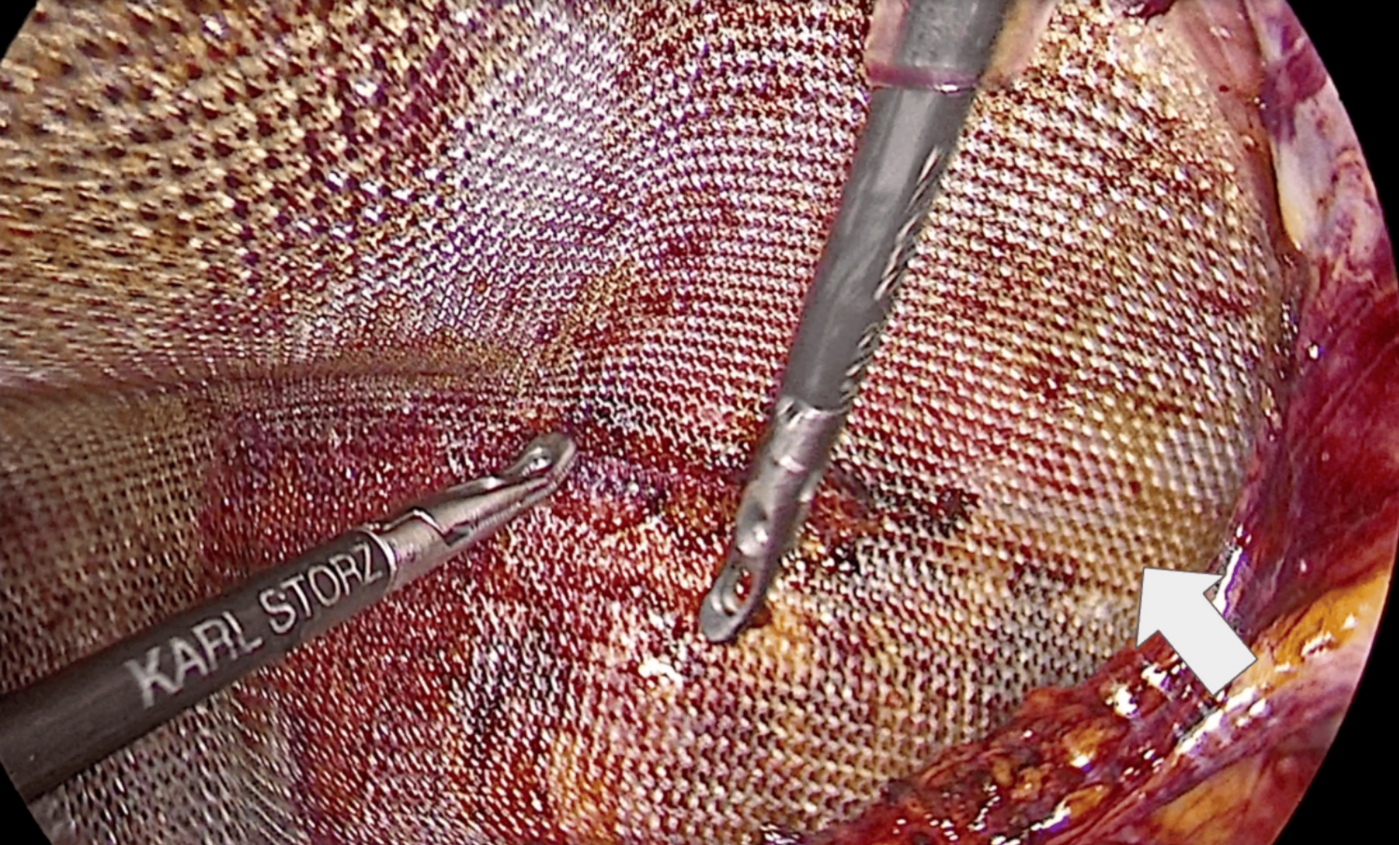
Bilateral prosthetic mesh placement within the preperitoneal space Intraoperative laparoscopic view following bilateral prosthetic mesh placement. The mesh is shown adequately expanded within the preperitoneal space, providing coverage of the hernia defects after reduction of the hernia trajectory and preperitoneal tissue (arrow).

Surgical management included reduction of the right-sided hernia defect and repositioning of the left preperitoneal fat. Bilateral prosthetic mesh placement was subsequently performed without complications. The mesh was adequately expanded within the preperitoneal space, achieving appropriate coverage of the hernia defects (Figure 3). No bleeding or vascular injury occurred, and mesh fixation was deemed adequate.

The postoperative course was favorable, consistent with the elective and minimally invasive nature of the procedure. At one-month follow-up, the patient remained asymptomatic, with normal gait and no evidence of recurrence.

## Discussion

The present case illustrates an exceptionally rare presentation of bilateral inguinal hernia with deep retroperitoneal extension toward the psoas muscle, identified as an incidental intraoperative finding. Inguinal hernia repair is performed worldwide at high volume, and bilateral defects represent a minority of cases; in female patients, such presentations are less frequent, further underscoring the rarity of the current finding [2,6-10].

When considered in conjunction with the deep retroperitoneal extension observed in the present case, these epidemiological factors further emphasize the clinical and anatomical singularity of this entity.

Literature review

To contextualize the anatomical uniqueness of the present case, a focused review of the literature was conducted to identify previously reported instances of inguinal hernias demonstrating deep retroperitoneal extension, psoas muscle involvement, or atypical posterior compartment defects encountered intraoperatively. Given the rarity and heterogeneity of these entities, the literature exploration was conceived as complementary to the case analysis rather than as a formal, systematic, or narrative review.

The search was performed primarily through PubMed/MEDLINE, complemented by Google Scholar to broaden case identification and capture reports not indexed in traditional biomedical databases. Publications between January 2005 and December 2025 were considered, limited to articles published in English and Spanish. Search terms combined Medical Subject Headings (MeSH) and free-text keywords related to inguinal hernia, retroperitoneal extension, psoas or iliopsoas involvement, para-psoas defects, retropsoas hernias, and incidental intraoperative findings during laparoscopic or endoscopic groin repair.

Initial database screening yielded a limited number of relevant publications, underscoring the scarcity of documented cases involving deep posterior extension. Additional manual review enabled identification of case reports and surgical series describing para-psoas, inguino-pelvic, retropsoas, prevascular, and para-inguinal hernia variants, as well as retroperitoneal pathologies mimicking inguinal defects. Anatomical systematization studies and contemporary groin hernia guidelines were also incorporated to support operative interpretation and anatomical orientation.

Collectively, the available literature confirms the exceptional rarity of inguinal hernias involving the psoas muscle or adjacent retroperitoneal compartments, with most diagnoses established incidentally during minimally invasive repair. A comparative synthesis of previously reported cases is summarized in Table 1.

**Table 1:** Comparative characteristics of reported atypical inguinal hernias with deep retroperitoneal or psoas-related involvement The table summarizes demographic features, anatomical subtype, clinical presentation, diagnostic performance of preoperative imaging, surgical approach, intraoperative findings, and postoperative outcomes. Most reported cases were diagnosed incidentally during laparoscopic repair, highlighting the diagnostic value of preperitoneal exploration in atypical groin pain. TEP: total extraperitoneal hernia repair; TAPP: transabdominal preperitoneal hernia repair; CT: computed tomography; MRI: magnetic resonance imaging; US: ultrasonography; FU: follow-up; n: number of patients; incl.: including

Study (year)	Hernia subtype	Patient demographics	Clinical presentation	Preoperative imaging diagnosis	Surgical approach	Hernia content	Intraoperative identification	Repair technique	Outcomes/follow-up
Badiani et al. (2021) [3]	Para-psoas	3 males (54-82 years)	Chronic inguinal pain/lumbar discomfort	Not identified	TEP	Preperitoneal fat	Incidental	Mesh repair (plug + preperitoneal mesh)	Complete resolution
Modeste and Novitsky (2012) [4]	Para-psoas/inguino-pelvic	42-year-old female	Chronic groin pain radiating to the thigh	Negative CT/MRI	TAPP	Incarcerated fat	Incidental	Preperitoneal mesh repair	Symptom resolution (two-year FU)
Goel et al. (2006) [5]	Psoas	26-year-old female	Reducible groin swelling + thigh pain	Not identified	TEP	Preperitoneal fat	Incidental	Mesh covering all defects	Complete resolution (six months)
Shenoy et al. (2022) [2]	Psoas	2 males (45-75 years)	Groin swelling + pain	Not identified	TEP	Preperitoneal fat	Incidental	Mesh fixation to the Cooper ligament	No recurrence (one-year FU)
Powell et al. (2015) [6]	Retropsoas	Series (n=3 within 2436 repairs)	Variable/often asymptomatic	Not identified	TEP	Fat/preperitoneal tissue	Incidental	Polyester mesh	Complete repair reported
Chen (2025) [7]	Para-psoas (appendiceal)	71-year-old female	Acute abdomen + chronic pain	Identified on CT	Laparoscopy	Appendix	Intraoperative confirmation	Appendectomy + defect closure	Favorable outcome
Veréb-Amolini et al. (2015) [8]	Para-inguinal	51-year-old male	Palpable mass	Identified on CT/US	TEP	Fat/bowel	Confirmed intraoperatively	Mesh repair	No recurrence (19 months)
Mourad and Kharbutli (2023) [9]	Para-inguinal	62-year-old female	Painful inguinal bulge	Identified on CT	TEP	Preperitoneal fat	Confirmed	Mesh repair	Seroma only
Matsevych et al. (2016) [10]	Multiple atypical (incl. prevascular)	68-year-old female	Persistent groin pain	Partial	TAPP	Multiple	Intraoperative	Bilateral mesh repair	Symptom resolution

Rarity of the condition and review of similar cases

Given the heterogeneity of published reports and the frequent absence of standardized reporting across case-based literature, we summarized the clinical presentation, imaging yield, and preoperative suspicion patterns across the included atypical groin hernia subtypes in Table 2. This synthesis highlights recurrent diagnostic themes relevant to surgical planning in patients with persistent groin pain and non-diagnostic imaging.

**Table 2:** Clinical and diagnostic features of atypical deep groin hernias identified in the included literature Data are summarized from the included case reports/series and one retrospective surgical series. Percentages are intentionally avoided in favor of n/N due to small, heterogeneous samples and incomplete reporting across studies. ^†^ No preoperative imaging was performed; the diagnosis was made intraoperatively during laparoscopic totally extraperitoneal (TEP) exploration.

Atypical hernia subtype	Typical presentation (from included reports)	Preoperative imaging performed	Atypical defect identified on imaging	Preoperative clinical suspicion of atypical hernia	Key clinical/diagnostic takeaway	Main sources (cases)
Para-psoas	Chronic/intermittent groin pain or discomfort; may radiate; often without a palpable mass; frequently found intraoperatively	2/5	2/2	0/5	Imaging may be negative or non-diagnostic in many; posterior laparoscopic/endoscopic exploration can reveal defects lateral to the psoas not appreciated clinically	Badiani et al. (n=3) [3], Modeste and Novitsky (n=1) [4], Chen (n=1) [7]
Psoas (intramuscular)	Groin pain with radiation to the thigh/hip; may present with groin swelling; typically diagnosed intraoperatively during TEP exploration	0/3^†^	NA	0/3	Often diagnosed only during systematic lateral dissection; it should be considered when symptoms persist despite “typical” hernia findings	Goel et al. (n=1) [5], Shenoy et al. (n=2) [2]
Retropsoas	Variable/unclear (largely described as rare intraoperative entities within a large surgical series)	Not consistently reported	Not consistently reported	Not consistently reported	Rare posterior defects may only be recognized through complete preperitoneal mapping; emphasis is on systematic exploration and anatomic recognition	Powell et al. (retropsoas n=3) [6]
Prevascular/retrovascular (femoral variants)	Often nonspecific; may be occult and discovered during laparoscopy; may coexist with other groin defects	Not consistently reported	Not consistently reported	Not consistently reported	Recognition prevents missed diagnoses or unexplained “recurrences”; requires deliberate assessment beyond classic myopectineal landmarks	Powell et al. (prevascular n=25) [7], Matsevych (n=1) [10]
Para-inguinal (lateral/interstitial)	Palpable mass ± pain; in some cases, identified preoperatively by imaging	2/2	2/2	1/2	Unlike deeper psoas-related defects, para-inguinal hernias may be more amenable to imaging detection; correct anatomic classification is crucial to avoid operative mis-targeting	Veréb-Amolini (n=1) [8], Mourad and Kharbutli (n=1) [9]

Overall, Table 2 underscores that many psoas-related and deep posterior variants are predominantly identified intraoperatively, whereas more superficial/lateral subtypes (e.g., para-inguinal defects) may be more readily suggested by preoperative imaging. These patterns support the role of systematic posterior compartment exploration during minimally invasive repair when symptoms persist despite negative cross-sectional imaging.

Bilateral inguinal hernias are recognized as technically more demanding than unilateral defects, often requiring advanced operative planning. Among an estimated 20 million inguinal hernia repairs performed annually worldwide, only about 10% occur in female patients, underscoring the demographic infrequency of this presentation. The surgical strategy employed in the present case aligns with contemporary recommendations favoring tension-free TAPP repair, which has demonstrated superior postoperative recovery, reduced chronic pain, and lower wound complication rates compared with open techniques [11].

While indirect inguinal hernias remain the most prevalent subtype, atypical sac contents have been described. Muscular involvement, most commonly internal oblique or transversus abdominis fibers, has been reported in only 0.19% of primary hernias and 0.81% of recurrent defects [12]. Beyond adipose tissue and bowel, unusual contents including cecum, appendix, fallopian tubes, and ovarian pathology have also been documented [13].

In contrast, psoas muscle involvement remains exceedingly rare and has been predominantly reported in male patients following pelvic surgery, particularly prostatectomy, suggesting a multifactorial interplay between fascial disruption and sarcopenia [14]. Comparable descriptions in elderly female patients are exceptionally scarce, reinforcing the clinical singularity of the present case [15-20].

Operative strategy and intraoperative detection patterns were further analyzed across reported cases, with particular focus on surgical approach and completeness of preperitoneal exploration in Table 3.

**Table 3:** Surgical strategies and intraoperative findings in reported cases of atypical deep groin hernias This table summarizes operative approaches, intraoperative detection patterns, and the feasibility of simultaneous repair across previously reported atypical groin hernia variants. TEP: totally extraperitoneal repair; TAPP: transabdominal preperitoneal repair; NA: not applicable or not reported

Hernia subtype	Surgical approach	Complete preperitoneal exploration	Incidental intraoperative diagnosis	Simultaneous repair performed	References
Para-psoas	TEP	Yes	Yes	Yes	[1,2,17]
Psoas	TEP	Yes	Yes	Yes	[14,15]
Retropsoas	TEP	Yes	Yes	NA	[16]
Prevascular/retrovascular	TAPP	No	Yes	Yes	[16,17]
Para-inguinal	TEP	Yes	No	Yes	[19,20]

Minimally invasive posterior approaches, particularly totally extraperitoneal (TEP), demonstrated consistent capability in identifying deep or retroperitoneal hernia variants not suspected preoperatively. Systematic preperitoneal dissection appears critical for accurate diagnosis and safe repair.

Atypical hernia contents and spectrum of unusual sac findings

Within the broad spectrum of inguinal hernia pathology, the majority of hernia sacs contain preperitoneal fat or small bowel loops, reflecting the classical pathophysiological mechanisms of groin herniation [1]. However, atypical sac contents have been increasingly documented in the literature, expanding the anatomical variability of inguinal defects. Reported unusual findings include cecum, appendix, sigmoid colon, urinary bladder, fallopian tubes, ovaries, and even neoplastic or inflammatory retroperitoneal structures [1,2]. Although rare, these presentations highlight the capacity of the inguinal canal and preperitoneal spaces to accommodate a wide range of visceral and parietal elements beyond traditional expectations.

Muscular involvement represents an even less frequent phenomenon. Prior reports have described herniation or protrusion of internal oblique and transversus abdominis fibers, with estimated incidences below 1% in both primary and recurrent repairs [3]. Such cases are often associated with posterior wall attenuation, fascial defects, or prior surgical disruption. In contrast, herniation involving deep retroperitoneal musculature, particularly the psoas muscle, remains exceedingly rare. Existing descriptions are limited to isolated case reports and small surgical series, most frequently identified incidentally during minimally invasive repair rather than through preoperative imaging [11,19,21].

The presence of psoas-related herniation, therefore, represents an extreme end of the anatomical spectrum of inguinal sac contents. This rarity underscores the importance of systematic posterior compartment exploration during laparoscopic repair and further reinforces the anatomical singularity of the present case within the existing literature.

Anatomical correlation and pathophysiological mechanisms

The psoas major muscle originates from the transverse processes and vertebral bodies of T12-L5 and courses retroperitoneally along the pelvic brim before merging with the iliacus to form the iliopsoas complex. Positioned posterior to the transversalis fascia and adjacent to the preperitoneal plane, it constitutes a deep anatomical boundary of the inguinal region and serves as the posterior floor of the preperitoneal space (Bogros space). Integrity of the posterior wall and deep inguinal ring is therefore critical in preventing protrusion of retroperitoneal structures [18].

Degenerative attenuation of the posterior inguinal wall, particularly in elderly individuals, may facilitate abnormal displacement of adjacent tissues. Although commonly described following pelvic surgical disruption, such as radical prostatectomy, similar structural weakening may arise through age-related fascial degeneration [17,22].

Sarcopenia represents an additional contributory factor. Radiologic indices of psoas muscle mass correlate with diminished abdominal wall integrity and adverse outcomes in abdominal wall reconstruction [23,24]. While direct causal evidence linking sarcopenia to psoas herniation remains limited, progressive muscular atrophy combined with fascial laxity provides a plausible mechanistic substrate for deep retroperitoneal protrusion.

Surgical implications and intraoperative considerations

Iatrogenic trauma to the psoas muscle or the adjacent femoral nerve during retroperitoneal dissection may result in bleeding, postoperative pain, hip flexion impairment, or neurovascular complications. Moreover, the proximity of the femoral nerve and iliac vessels elevates the risk of neurovascular injury during retroperitoneal dissection.

Consequently, careful reduction rather than resection of muscular content is recommended, followed by wide preperitoneal mesh reinforcement. Adequate coverage of the myopectineal orifice remains essential to prevent recurrence, particularly in the presence of posterior wall attenuation [20]. Recognition of unusual content should therefore prompt meticulous anatomical dissection and individualized operative strategy.

Comparison with published literature and clinical impact

The present case aligns with prior reports describing occult inguinal hernias undetectable on preoperative imaging yet identified during laparoscopic exploration [3,4,18]. However, the patient’s advanced age exceeds that reported in most published cases, suggesting that deep retroperitoneal extensions may remain clinically silent for prolonged periods until degenerative muscular changes permit symptomatic protrusion [2].

From a diagnostic standpoint, negative cross-sectional imaging should not exclude hernia pathology in patients with persistent inguinal pain. Laparoscopic exploration, particularly via TAPP, provides superior visualization of preperitoneal and retroperitoneal compartments, supporting its dual diagnostic and therapeutic value [6]. Recognition of psoas-related extensions broadens the anatomical spectrum of occult hernias and reinforces the importance of posterior compartment evaluation.

Limitations and contributions to surgical knowledge

This report is limited by its single-case design, short follow-up duration, and the absence of dedicated preoperative imaging specifically evaluating deep retroperitoneal planes, which restricts comprehensive radiologic correlation and long-term outcome assessment.

Nonetheless, it contributes meaningful insight into the expanding spectrum of atypical inguinal hernia presentations. The case highlights the potential for deep retroperitoneal muscular involvement, underscores the diagnostic utility of laparoscopic exploration, and reinforces the importance of advanced anatomical knowledge when managing occult groin pathology. Recognition of these rare variants may enhance intraoperative preparedness, reduce iatrogenic risk, and refine surgical planning in complex hernia repair.

## Conclusions

Bilateral inguinal hernia with deep retroperitoneal extension toward the psoas muscle represents an exceptionally rare anatomical variant that may remain undetected during preoperative assessment. The present case highlights the intraoperative identification and successful laparoscopic management of asymmetric posterior extension in an elderly female patient, underscoring the diagnostic limitations of routine imaging in atypical groin pathology and its potential impact on surgical planning. When contrasted with the available literature, this presentation remains infrequently documented, particularly given its bilateral nature and deep posterior compartment involvement, thereby expanding the currently recognized morphological spectrum of inguinal hernia disease.

From a surgical standpoint, recognition of occult posterior extension is critical to prevent neurovascular injury, uncontrolled bleeding, postoperative pain, functional impairment, and incomplete repair. Systematic exploration of the posterior myopectineal region during minimally invasive approaches facilitates individualized operative planning, safe reduction of herniated contents, and adequate mesh coverage. Clinically, persistent or disproportionate groin symptoms in the setting of negative imaging should raise suspicion for concealed retroperitoneal involvement. Favorable short-term postoperative outcomes without early recurrence support the safety and effectiveness of laparoscopic management when meticulous anatomical assessment is performed, although long-term surveillance remains essential. Ultimately, this report reinforces the importance of heightened intraoperative vigilance, refines surgical awareness of complex posterior variants, and provides educational value that may inform future case accumulation and operative strategy standardization for atypical inguinal hernia presentations.

## References

[1] Rosenberg J, Baig S, Chen DC, Derikx J (2025). Groin hernia. Nat Rev Dis Primers.

[2] Shenoy GK, Kulkarni AA, Makam R (2024). Endoscopic diagnosis and management of occult atraumatic psoas hernia. Indian J Surg.

[3] Badiani S, Cooper EA, Diab J, Berney CR (2021). Occult para-psoas hernia during routine endoscopic totally extraperitoneal inguinal hernia repair. JSLS.

[4] Modeste K, Novitsky YW (2013). Laparoscopic diagnosis and management of a novel inguino-pelvic hernia. Hernia.

[5] Goel A, Soni V, Khullar R, Sharma A, Baijal M, Panse R, Chowbey PK (2006). A rare psoas hernia: endoscopic diagnosis and management. Surg Laparosc Endosc Percutan Tech.

[6] Powell BS, Lytle N, Stoikes N, Webb D, Voeller G (2015). Primary prevascular and retropsoas hernias: incidence of rare abdominal wall hernias. Hernia.

[7] Chen Z (2025). Rare appendicitis with para-psoas appendix hernia: a case report. J Surg Case Rep.

[8] Veréb-Amolini L, Betschart T, Kiss E, Ullrich O, Wildi S, Eppler E (2015). An atypical lateral hernia and concomitant inguinal and umbilical hernias in a patient with polycystic kidney disease and an intracranial aneurysm - a combined approach of clinical and radiological investigation, endoscopic hernia repair, and anatomical cadaver model documentation and a systematic review of the literature. Springerplus.

[9] Mourad M, Kharbutli B (2023). Para-inguinal hernia; presentation, diagnosis and surgical treatment, a case report. Int J Surg Case Rep.

[10] Matsevych OY, Koto MZ, Becker JH (2016). Multiple concurrent bilateral groin hernias in a single patient; a case report and a review of uncommon groin hernias: a possible source of persistent pain after successful repair. Int J Surg Case Rep.

[11] Oliveira Carneiro A, Rocca WR, Gonçalves LB, Mazzola Poli de Figueiredo S (2026). Laparoscopic totally extraperitoneal (TEP) versus laparoscopic transabdominal preperitoneal (TAPP) for bilateral inguinal hernia repair: a systematic review and meta-analysis. Hernia.

[12] Szasz P, Mainprize M, Spencer Netto FA (2023). Muscular groin hernias: an anatomical variation as a cause of recurrence. Hernia.

[13] Chauhan R, Saurabh A, Yadav V (2021). Inguinal hernia containing hemorrhagic ovarian cyst in an adolescent: a rare case report. Int J Abdom Wall Hernia Surg.

[14] Fukata S, Shimasaki S, Yamashita E (2025). Efficacy of preventive techniques against postoperative inguinal hernia in patients who underwent robot-assisted radical prostatectomy for Japanese patients. J Robot Surg.

[15] Ungureanu CO, Ginghina O, Stanculea F (2023). Surgical approach to bilateral inguinal hernia. A case-control study. Chirurgia (Bucur).

[16] Agarwal PK (2023). Study of demographics, clinical profile and risk factors of inguinal hernia: a public health problem in elderly males. Cureus.

[17] Alder R, Zetner D, Rosenberg J (2020). Incidence of inguinal hernia after radical prostatectomy: a systematic review and meta-analysis. J Urol.

[18] (2018). International guidelines for groin hernia management. Hernia.

[19] Clementi M, Di Furia M, Sista F, Mackay AR, Guadagni S (2020). Successful laparoscopic trans-peritoneal repair of an incisional inguinal hernia, resulting from deep lymph node dissection for melanoma: a case report. Int J Surg Case Rep.

[20] Zhu M, Liu Y, Lou Y, Ma C, Gu J, Ren L (2023). Analysis on the occurrence and risk factors of inguinal hernia after radical prostatectomy with different surgical methods. J Clin Urol.

[21] Otaki T, Hasegawa M, Yuzuriha S (2021). Clinical impact of psoas muscle volume on the development of inguinal hernia after robot-assisted radical prostatectomy. Surg Endosc.

[22] Yamada Y, Fujimura T, Fukuhara H (2017). Incidence and risk factors of inguinal hernia after robot-assisted radical prostatectomy. World J Surg Oncol.

[23] Bailey CM, Schaverien MV, Garvey PB, Liu J, Butler CE, Mericli AF (2020). The impact of sarcopenia on oncologic abdominal wall reconstruction. J Surg Oncol.

[24] Santana Valenciano Á, Blázquez Hernando L, Robín Valle de Lersundi Á (2024). Role of sarcopenia in complex abdominal wall surgery: does it increase postoperative complications and mortality?. Hernia.

